# Need for and relevance of prostate cancer screening in Nigeria

**DOI:** 10.3332/ecancer.2014.457

**Published:** 2014-08-28

**Authors:** TO Akinremi, A Adeniyi, A Olutunde, A Oduniyi, CN Ogo

**Affiliations:** 1 Department of Pathology, Federal Medical Centre, Idi-Aba, Abeokuta 110001, Nigeria; 2 Department of Surgery, Federal Medical Centre, Idi-Aba, Abeokuta 110001, Nigeria

**Keywords:** digital rectal examination (DRE), African American (AA) men, Nigerian men, prostate-specific antigen (PSA), prostate cancer (PCa), screening

## Abstract

Prostate cancer (PCa) has become the most prevalent cancer among males in Nigeria, and similar to other black populations, Nigerian men present with more advanced disease at an earlier age than in several other ethnic groups. In this unscreened, high-risk group, the reference range for early detection and diagnosis as well as risk factors need to be determined through large-scale screening. Over 4 years, 1124 previously unscreened men between 40 and 85 years of age were screened at free community health programmes for PCa, using the common parameters of prostate-specific antigen (PSA) plus digital rectal examination (DRE). We thereby assessed the practicality and importance of screening. Consent was obtained, demographic data obtained, PSA measured using qualitative laboratory kits, and DRE performed by surgeons.

We found that the number of men attending and consenting to screening increases from year to year. Of 40–85-year-old men, 85.4% consented, of whom 33.3% (a third) and 60% were 51–60 years old and 51–65 years, respectively. While 11.5% of men had PSA >4 ng/ml, 31.45% showed abnormal DRE. Of the men who took the PSA test, 79.2% also consented to the DRE, of whom 5.8% had combined abnormal DRE and PSA >4 ng/ml.

Our findings suggest that Nigerian men are a willing group for screening by both the PSA and DRE with the positive response to calls for health screening and interest in prostate health. The finding of PSA >4 ng/ml in 11.15% of this population reveals the need for greater awareness and measures to increase early detection. However, the value and validity of established PSA reference ranges and cutoff of ‘normal’ still need to be established. Screening is very important to better define the PCa prevalence and characteristics in our population; otherwise political and economic circumstances will ensure that men still present late with aggressive PCa.

## Introduction

With regards to prostate health, the Nigerian male population is an unscreened group. Increasingly, the literature shows that prostate cancer (PCa) is on the rise among Nigerian men just as is being noted in other men of African descent in developing as well as in developed countries. It is also often aggressive and discovered at later stages. Rebbeck *et al* [1] observed that lower rates of PCa in Africa may in part reflect a lower probability of PCa detection than in countries like the US where prostate-specific antigen (PSA) and digital rectal examination (DRE) screening have been more widely used. The authors also commented that African-specific risk profiles may require different screening and treatment strategies in Africa than those in standard practice in North America or Europe.

There are as yet many unknowns about PCa risk factors specific to the black race generally, and though some similarities have been noted between African American (AA) men and native African men, there are also discordances. For example, PCa is thought to be more prevalent among AA than African men. But, is this really correct [2]? We do not know because of a dearth of research data in Africa, Nigeria inclusive. The comparison is therefore impossible. Research into trends, prevalence, incidence, and morbidity/mortality of PCa is limited as well as that for genetic and other risk factors. So far, little is known about the value of the current major marker for PCa, the PSA, and its applicability and practicability for screening in the Nigerian male populace. ‘Normal’ and age-specific ranges of PSA have not been determined.

Awareness, education, and screening as prerequisites for cancer prevention are quite low in the developing world, partly, but very importantly, because of a lack of political will of the leaders that are inadvertently expected to initiate the process, and the seemingly overarching need to face communicable diseases [3].

Screening for a disease focuses on finding ‘markers’ of the disease in an otherwise healthy person or population in order to arrive at an early detection of the disease. This is of especial importance in cancer detection. PCa screening via the PSA test has been utilized in developed countries for years, enabling building of the background for groundbreaking research that is improving patient care now [1].

Community outreach for health purposes utilizes invitations to the community via educational billboards, flyers, mass media, lectures, video and so on, as well as screening for other non-communicable diseases during which time awareness can be raised for both men and women, adults and youth.

The specific aim of this study was to investigate whether screening is acceptable by Nigerian men as well as to analyse the relevance of the results of screening among some Nigerian men over a period of 4 years (2005–2008).

## Materials and methods

Ethical approval was obtained from the Ethical Review Committee of the Federal Medical Centre (FMCA), Abeokuta, Nigeria, to collect bio-data and blood samples as well as to conduct DREs on consenting men 40 years and above over a period of four years (2005–2008). The location, Abeokuta in Ogun State, Nigeria, is situated on the east bank of the Ogun River with a population of about 449,088 [4].

Invitations were sent out through handbills and posters as well as through the news media and churches. Prostate health education was provided to both men and women, young and old, after which free a health screening, including for non-communicable diseases like diabetes and hypertension were performed. Prostate screening involved collection of venous blood followed by DRE. PSA was processed on-site with qualitative screening kits using a cutoff point of 4 ng/ml as significant. Referrals were made to the surgeon for follow up for PSA >4 ng/ml and/or abnormal DRE. The data were subjected to simple statistical analysis.

## Results

**Figure 1.**
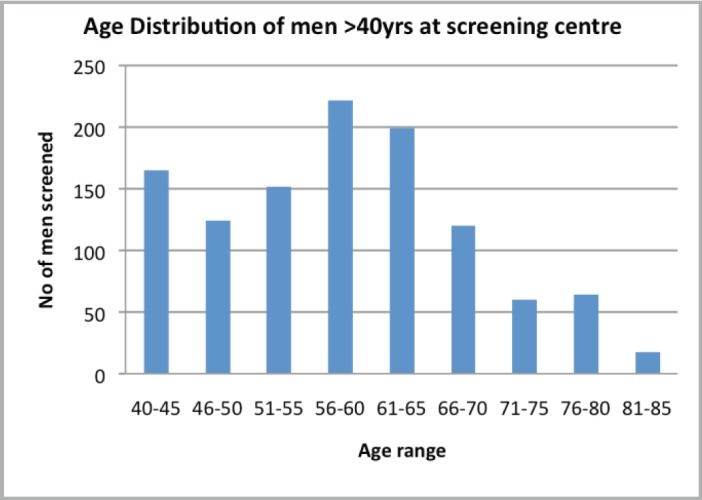
Age distribution of men >40 years of age who were screened.

**Figure 2.**
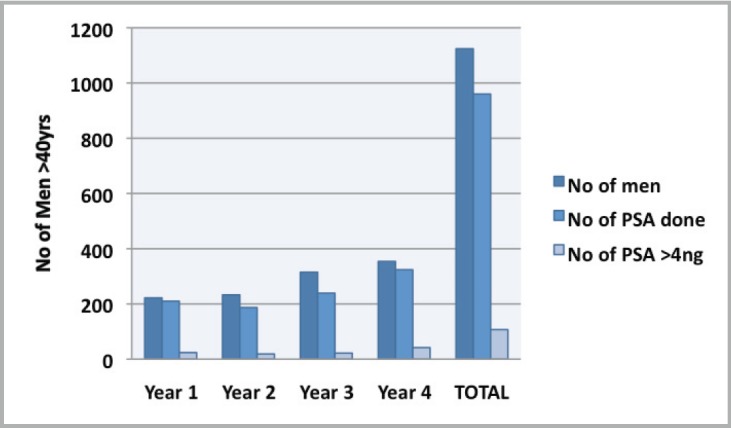
Trend of PSA in men >40 years of age by year of screening.

**Figure 3.**
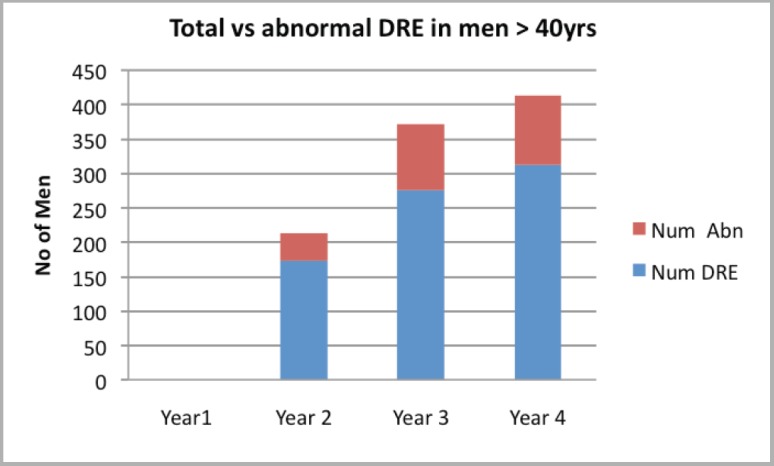
Proportion of abnormal DREs to total DREs.

Attendance was good at all the outreach events and increased from year to year (Table 1). Of the 1,292 male attendees, 1,128 men (87.3%) were 40 years or older, with a peak between 56 and 65 years. PSA testing was carried out on 85.4% (960) of men ≥40 years who consented to screening. The age distribution is as shown in Figure 1, with 33.3% and 60% of screened men being between 51 and 60 years and 51 and 65 years, respectively. Of the screened men, 11.15% had PSA ≥4 ng/ml (Figure 2). DRE was not done in the first year. In subsequent years, 77.6% of men had the DRE, of which 31.4% showed some abnormality (Figure 3), ranging between various degrees of enlargement and nodularity. Of all men examined, 3.9% had both an abnormal DRE and PSA >4 ng/ml.

## Discussion

Poor education about cancer amounts to exposing the populace to misinformation and can be more dangerous than no information. Health screening is an avenue for educating people, hence the need for organised screening. Even in developed countries, PCa disparity studies show that minority ethnic groups like AA men with poor awareness and screening are high-risk groups [5], whereas it seems they could have benefited from education and screening earlier because some risk could have been elucidated. Thankfully, Nigerian men, as exemplified by this screened group, show the willingness and eagerness to be screened following adequate information.

In Nigeria today, statistical data vary as to the prevalence of PCa. Following reports from various parts of the country, in 1999 Ogunbiyi and Shittu [6] from the Ibadan Cancer Registry announced a definite increase of PCa among Nigerians: it rose from eighth position in 1969 to first position in 1996, accounting for 11% of all male cancers. Since then, many more studies from different geographical zones in Nigeria have reported increasing prevalence. Kano [7] (north-west), Zaria (north-east) [8], Benin (south south) [9], and Lagos (south-west) [10] showed PCa prevalence as 16.5%, 9.2%, 7.13%, and 9.92%, respectively, of male cancers. The highest mortality from PCa was also reported in most of these studies with recent preliminary data from a five-year cohort study revealing that 29 out of 128 cases of PCa who presented at the FMCA Abeokuta with advanced disease were all dead within two years [oral communication].

The results of a small PCa screening initiative among 200 previously untested rural Nigerian males demonstrated that their 14% incidence of PSA levels ≥4 ng/ml was comparable to that of previously unscreened high incidence populations, such as AA men [11]. That study recommended the need for a PCa awareness and education campaign in Nigeria.

Most reports of PCa research reveal a lot of prospective or established risk factors in most populations, but these have been done in developed countries. Prostate cancer disparities focusing on AA men have directed researchers to look at the relevance of the transatlantic slave trade and thereby the possible impact of genetic and environmental risk factors [5]. Biorepositories are already being established to enhance transatlantic studies that will answer some PCa disparities questions. It is important to note that a major source of biorepository material and data is screening. Apart from ascertaining epidemiology of PCa and relevant ranges for PSA, translational research and personalised patient care will be made possible through screening. This will benefit developing countries too, as more data are harvested in clinical as well as screening settings. The global focus on PCa disparities in black men calls for more efforts from Africa, in all areas of research, along with international collaborations for capacity building [12].

Recent calls from various international professional advisory and regulatory bodies [13, 14] for modifications in screening practices in developed countries will impact activities in minority groups including the developing countries if care is not taken. If these recommendations from developed countries are followed only by other developed countries, it will leave significant (population specific) knowledge gaps that developed countries had achieved in years past by screening. It is, however, quite settling to see that the recommendations for and against screening are not absolute and that the overarching call is for risk-adapted action [15]. In this way, individuals, groups, and communities can demand for screening plans that suit their risk assessment [16]. It is expected that the Nigerian Urological Association and the Nigerian Cancer Society will come up with recommendations soon to give direction in respect of screening. No Sub-Saharan African country has done so yet, and screening activities are still ad hoc. Nigeria presently has no national screening programme for PCa, but opportunistic screening is on the rise for all cancers, and this is expected to positively feed the National Cancer Registry [10].

## Conclusion

The rising trend in Nigeria of PCa prevalence and risks calls for concerted efforts from all stakeholders including government and non-governmental organisations to regulate the processes of early cancer detection and prevention. Education, information, and screening programmes must be more organised, and central reporting of activities should be enabled. It is a good thing that Nigerian men are positive responders for screening, which is more relevant and urgent in our previously undefined population.

One of the best ways we would like to advocate for disseminating information about PCa screening is through community engagement and advocacy. During two of the outreach events, t-shirts with the logo ‘ASK ME’ in front and ‘About Prostate Cancer’ at the back were distributed to screened men. These men become advocates, especially if they are community leaders. A community-based video presentation has been developed to demonstrate answers to common questions about PCa. It is expected that following these sort of community activities, more men will voluntarily request screening.

## Conflicts of interest

The authors declare that they have no conflicts of interest.

## Figures and Tables

**Table 1.** Attendance of men at outreach events.

	Year 1	Year 2	Year 3	Year 4	Total
Number of males	267	271	356	394	1,288
Number >40 years	222	233	315	354	1,124
Number PSA done	210	187	239	324	960
Number PSA >4 ng/ml	24	19	22	42	107
DRE done		173	275	312	760
Abnormal DRE		40	97	102	239
